# Gender Gap in the Leadership of Health Institutions: The Influence of Hospital-Level Factors

**DOI:** 10.1089/heq.2021.0013

**Published:** 2021-08-13

**Authors:** Soledad Pérez-Sánchez, Sara Eichau Madueño, Joan Montaner

**Affiliations:** ^1^ Stroke Unit, Neurology Department, Hospital Universitario Virgen Macarena, Sevilla, Spain.; ^2^ Neurovascular Research Group, Biomedicine Institute of Seville, Sevilla, Spain.; ^3^ Multiple Sclerosis Unit, Neurology Department, Hospital Universitario Virgen Macarena, Sevilla, Spain.

**Keywords:** gender gap, management positions, health care institutions

## Abstract

**Objective:** To analyze whether the increased representation of women in the health field is accompanied by a greater presence in leadership positions in the public health system and whether there are differences according to the hospital level.

**Methods:** A descriptive study of the distribution of leadership positions by sex and type of hospital within the health centers of a regional public health system.

**Results:** In total, 74.01% of the professionals were women. The representation of women in management positions was 33.1%, and among service chiefs, it was 24.01%. In the service headings, we observed that surgical specialties had a lower representation of women (30.9% in medical specialties vs. 18.1% in surgical specialties, *p*<0.0001). By type of hospital, no differences were found in the management positions, but there were differences in the medical chiefs, with less female representation in the regional hospitals (28.6% vs. 39.7%, *p*=0.003).

**Conclusion:** Women represent the majority in the public health system. Nonetheless, their representation in positions of greater responsibility and decision-making is very limited, being particularly low in county hospitals. Increasing female representation in these positions is a current challenge for society, and equality policies need to be developed and applied to minimize this gender gap.

## Introduction

The representation of women in the health field is growing and exceeds that of men in this sector. In medicine, the percentage of women increased from 36.8% in 2000 to 54.3% in 2016 according to data from the Spanish National Health System Report of 2018.^1^ Despite this, the representation of women in key health sectors is deficient, limiting their capacity to influence or make decisions regarding health policies in academic institutions, scientific societies, or large clinical research centers.

The increase in women's presence in a profession does not always lead to a feminization of the positions of responsibility. The well-known glass ceiling and the so-called leaky pipeline are phenomena that underlie this fact. The former refers to the invisible barriers to the promotion of women in their professional careers, and the latter refers to the disappearance of women on the higher rungs of these careers.^2^ Behind this underrepresentation are gender inequalities, such as domestic and family burdens, stereotypes, and inequalities of opportunity.^3^

For all these reasons, our objective is to analyze the distribution by sex in leadership and decision-making positions in the health administration of our environment.

## Materials and Methods

A descriptive study of the distribution by sex in the leadership positions of regional public health service hospitals was carried out. The information was requested through the Transparency Portal of the government, and the data provided are as of April 30, 2019.

Data were obtained on the total number of workers and their distribution by sex. Leadership roles were divided into three large groups: management positions, medical chiefs (head of service and clinical chiefs), and nursing chiefs (block chief and supervisor). Among the management positions were managing directors, deputy managing directors, medical directors, deputy medical directors, economic administrative directors, economic administrative deputy directors, nursing directors, and deputy nursing directors. The analysis of medical positions was also carried out by specialty (medical or surgical).

In addition, a comparative study of these positions was carried out according to the type of hospital: regional and nonregional.

## Results

The total number of workers in public hospitals was 62,946 people, and 74.17% were women. The health care staff comprised 43,351 workers (68.87%), with 33,873 women (78.14%) and 9478 men (21.86%). The rest of the employees were from administration and services. Women accounted for 52.2% of the specialist medical staff and 77.9% of the nursing staff.

In terms of management positions, 58.27% of all positions were held by men. Overall, the probability of a man achieving one of these positions is 3.85 times higher than that of a woman (OR 3.85, 95% CI 3.02–4.92). Among the management and submanagement positions, men represented 64.29% and 55.63%, respectively. Among the director-general and economic-administrative positions (including subdirectorates), men's representation was higher, with men occupying 76.9% of these positions. Among the medical and nursing directorates and subdirectorates, men represented 55.56% and 45.83%, respectively.

**FIG. 1.**
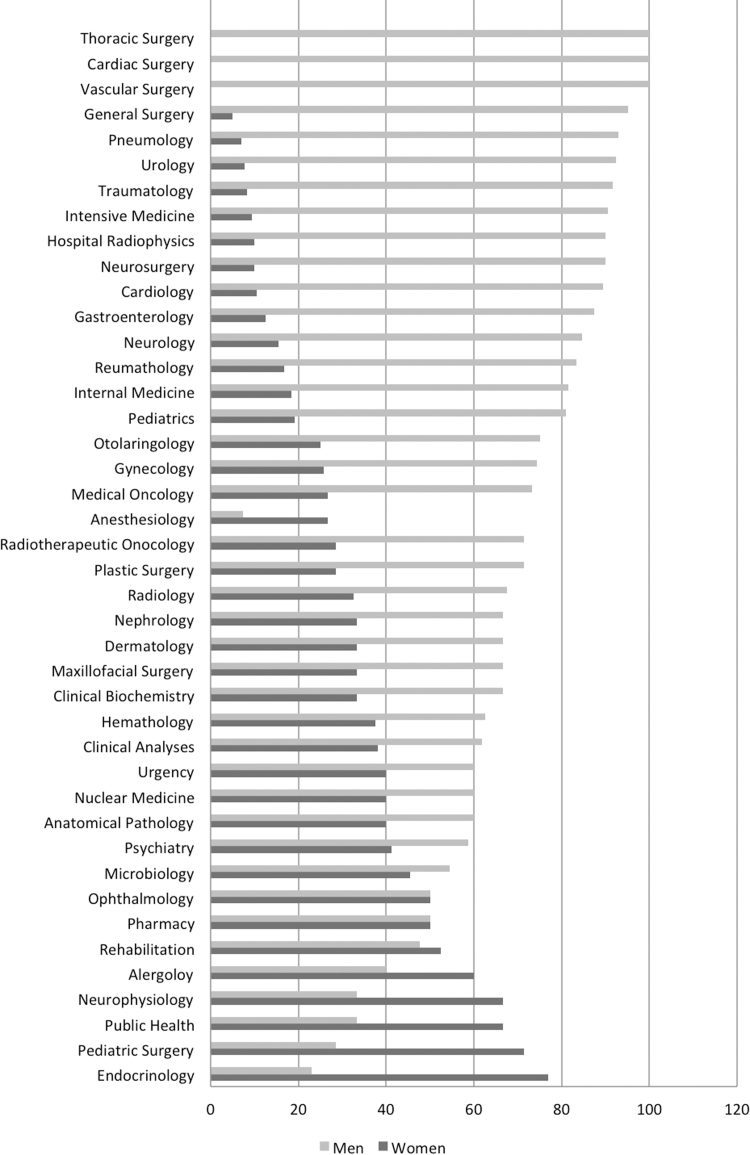
Distribution of medical positions by sex (%) and specialty.

In the medical positions referred to as service and section chiefs, we found that 72.55% of the total were occupied by men. They are still more likely to occupy these positions than women (OR 3.61, 95% CI 3.08–4.23). The complete distribution by sex and specialty is represented in Figure 1. Among the service chiefs, the representation of women was 24.01%, and this percentage was higher among the section chiefs (30.28%). According to the type of specialty, we found that in the surgical specialties, there was a lower representation of women overall (30.9% in medical specialties vs. 18.1% in surgical specialties, *p*<0.0001). Although this proportion was significantly inverted in the case of section chiefs (17.7% in physicians vs. 34.3% in surgeries, *p*<0.001), no differences were found in the case of service chiefs.

When analyzing the nursing positions as a whole (block chief and superintendent), the representation of men was 31.5%. Women held 66.7% of block chief positions and 68.85% of supervisory positions. Despite these percentages, men continue to be more likely than women to hold management positions (OR 2.12, 95% CI 1.78–2.52).

**FIG. 2.**
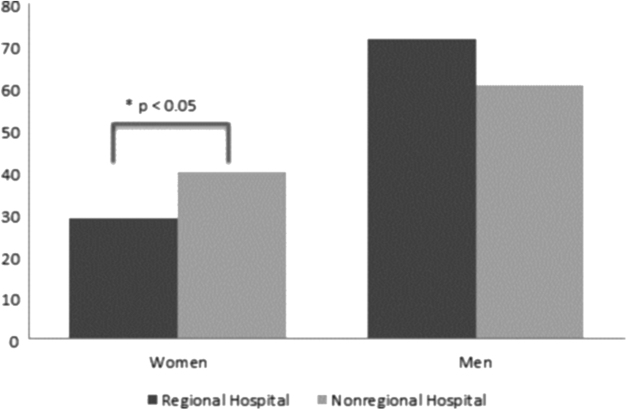
Distribution of medical positions by sex (%) and type of hospital.

By type of hospital, there were no differences in the distribution by sex among the management positions. The distribution of medical posts (head of department and section) by type of hospital is shown in Figure 2. In this study, we found a lower representation of women in regional hospitals (28.6% in regional hospitals vs. 39.7% in the rest of the hospitals, *p*=0.003). However, when analyzed separately, this relationship is reversed, with higher female representation among the service heads of regional hospitals (30.7% in regional hospitals vs. 19.6% in the rest, *p*=0.018). No significant differences were found for section chiefs. There were no differences between the two types of hospitals with regard to nursing command posts.

## Discussion

The data show that the phenomenon of the “leaky pipeline” is a reality in health institutions, since as it escalates in management positions, female representation decreases. Thus, we find that the representation of women is lowest in positions of greater responsibility, such as management, economic/administrative management, and service and block chief positions. This phenomenon has already been pointed out in other sectors and affects all areas of society globally.^4^ In the health sector, women occupy fewer representative positions in scientific societies and in collegiate bodies. In the academic or research field, although the number of women is growing, it has been observed that women's publications and participation in panels of experts or in the management of scientific centers is still lower.^5,6^

The gender disparity in health care leadership is pronounced. Globally, it is estimated that women represent 70% of health workers, yet only 26% of representative positions are held by women.^6^ In our study, women appear more often in subdirectorates than in directorates and are more often section chiefs than service chiefs. This fact is especially striking considering that women represent more than half of the medical personnel hired. Arrizabalaga et al. noted that compared with two-thirds of male doctors, only one-third of female doctors reached the highest positions in their careers.^5^

Historically, nursing has been considered a female-dominated profession. The idea of being a “good nurse” requires being “a good woman,” introduced by Florence Nightingale in the 19th century is based on the idea that nursing is perceived as women's work assuming that a woman's function is to care for and protect others.^7,8^

Although the proportion of male nurses has increased, it is still only 5–10% of the total of the nursing workforce in the western world, nursing continues to be seen as a female profession and thus its leaders are more often women; however, the percentage does not reach the same level as the percentage of female workers.^9,10^

With regard to the type of specialty, our study is limited because we do not have the distribution of personnel by type of specialty available to calculate ratios of professionals by sex against intermediate positions for each of the services. However, the data reported matched our observations and showed a clear masculinization of surgical specialties as opposed to medical specialties.^11^ The belief that a male surgeon is more reliable or factors encouraging women to choose nonsurgical specialties have been noted as reasons underlying this fact. In addition, the penalty for maternity is higher for female surgeons than it is for male surgeons, such that a lower percentage of female surgeons have children.^8^ Among the medical specialties, we found a higher proportion of women, especially in family and community medicine. Other traditionally masculinized specialties, such as cardiology or neurology, are changing and have reached greater feminization, but they are still far from matching women's overall representation.^12,13^

The type of hospital does not seem to affect gender inequality in the leadership of health centers, either,^14^ and in fact, in our study, the differences are maintained or even increased in regional hospitals, as in the case of a lower proportion of women among the clinical heads (despite a greater number of female heads of service). These differences could also be related to the greater power that this type of hospital holds within the administration as a whole. In this section, it is important to know that the distribution of service and section heads is very unequal between the different hospitals and could influence the results.

The causes for women's lack of representation in these positions are varied and are due to gender inequalities, such as family burdens, inequality of opportunity, or lack of mentors.^5,15^ The possibility of mentoring and sponsorship, as well as the creation of and participation in networks, are turning points in the development of professional careers and are not always available for women.^16^ In this respect, initiatives for the participation of women at all levels of leadership, such as the one carried out by the European Stroke Organisation (Women's Initiative for Stroke in Europe—WISE), are achieving positive results in visibility and promotion.^17^ Although one might think that it is women who do not want to access leadership positions in their work, a survey of 1200 female doctors about their experience and interest in leadership positions revealed that 60% of them would consider running for a leadership position in their workplace, and ∼50% would run for a position in their medical society in the future. Family time and lack of encouragement from their bosses were the greatest obstacles they faced.^18,19^ A survey of Andalusian health service professionals revealed that majority of women most frequently state that they do not want to be promoted due to family circumstances. It is also interesting to note in this survey regarding the perception of equality among professionals. Thus, a higher percentage of men (more than a half), in contrast to women, consider that it is not necessary to implement equality plans. This perception is even higher among basic and intermediate positions.^20^ These differences in leadership positions also have consequences for the salary gap, with the health sector having the greatest difference between men and women, according to the Spanish National Institute of Statistics.^21^

Minimizing these inequalities is a challenge for society, and institutions must promote and facilitate women's leadership capacity. Interventions aimed at facilitating motherhood and reconciliation, encouraging and establishing scenarios that allow for women's promotion, avoiding stereotypes and situations of harassment or discrimination, or even reserving leadership positions for women, will make it possible to reduce the current gender gap in the health field.

## Abbreviations Used

CI: confidence interval
OR: odds ratio
WISE: Women's Initiative for Stroke in Europe
